# Associations between serum estradiol and IL-6/sIL-6R/sgp130 complex in female patients with major depressive disorder

**DOI:** 10.1186/s12888-023-05248-z

**Published:** 2023-10-12

**Authors:** Ting Sun, Qian Chen, Junchi Mei, Yan Li

**Affiliations:** Department of Clinical Laboratory, Renmin Hospital of Wuhan University, Zhangzhidong Road, Wuchang District, Wuhan, 430060 Hubei China

**Keywords:** Major depressive disorder, Estradiol, Soluble glycoprotein 130, Soluble interleukin-6 receptor α, Interleukin-6

## Abstract

**Background:**

It has been hypothesized that the IL-6/sIL-6R/sgp130 complex, an inflammatory complex, plays a critical role in the pathogenesis of major depressive disorder (MDD). Estradiol (E2) is a sex steroid hormone involved in emotional regulation and MDD. This study aimed to investigate the relationship between E2 and IL-6/sIL-6R/sgp130 complex in patients with MDD.

**Methods:**

Using enzyme-linked immunosorbent assay, the levels of IL-6, sIL-6Rα, and sgp130 were compared between 117 female patients with MDD and 122 healthy controls.The serum concentrations of E2 and other biomarkers were also measured.

**Results:**

(1) The serum levels of IL-6 and sIL-6Rα in patients with MDD were significantly higher than those in the control group, while the serum levels of sgp130 and E2 were significantly lower (all *P* < 0.05). (2) Low levels of E2 were associated with high levels of IL-6 and low levels of sgp130 (all *P* < 0.01). (3) HAMD-24 score was positively correlated with the serum level of IL-6, but negatively correlated with the serum levels of sgp130 and E2(all *P* < 0.05). (4) IL-6 and sgp130 had certain prognostic values in MDD, and the combination of various indicators showed a significantly superior prognostic value.

**Conclusions:**

The IL6/sIL-6R/sgp130 complex in female patients with MDD was closely related to E2 level. In addition, IL-6 and sgp130 may be valuable serum biomarkers for the diagnosis and prognosis of MDD in women.

## Introduction

Major depressive disorder (MDD), a common public health problem, is the most frequent cause of disability and death [1, 2]. MDD not only brings heavy mental and economic pressure to patients but also imposes a great burden on families and society. However, the importance of MDD has not been fully understood, and most patients do not receive timely treatment. Therefore, it is important to explore the potential mechanisms and variable risk factors triggering MDD.

Inflammation, as a common feature of mental illness, is a vital factor in the pathogenesis and exacerbation of MDD [3]. Increased levels of peripheral inflammatory markers are common findings in MDD and inflammation. Interleukin 6 (IL-6) is the most common inflammatory cytokine, which plays a significant role in the pathogenesis of MDD. Recently, several studies highlighted that elevated levels of IL-6 are closely related to MDD [4, 5]. IL-6 binds to the soluble IL-6 receptor (sIL-6R) to form an IL-6/sIL-6R complex, which then binds to the membrane binding glycoprotein 130 (gp130) subunit, transduces trans-signaling, and triggers a pro-inflammatory response [6]. It has been hypothesized that IL-6 trans-signaling is closely associated with MDD, cancer, and chronic inflammation [6, 7]. Ferencova et al. [8] reported that MDD is a chronic inflammatory disease that can activate the inflammatory response system (IRS) and elevate the serum levels of IL-6 and sIL-6R levels. Animal studies have also shown that IL-6 trans-signaling is associated with “disease behavior” similar to depression, and inhibition of IL-6 trans-signaling alleviated pathogenic behavioral symptoms [7]. In addition, it has been found that soluble glycoprotein 130 (sgp130) can competitively bind to the IL-6/sIL-6R complex, thereby preventing trans-signaling [9, 10]. Sgp130 has been extensively studied in pancreatic cancer [11], inflammatory acute lung injury [12], atherosclerosis [13], and other diseases, while its role in mental diseases, such as depression, is poorly understood. Therefore, this study measured the serum levels of sgp130 in patients with MDD.

MDD is an emotional disorder related to sex hormones.Changes in estrogen levels are particularly important in female patients with MDD. Estrogen, a steroid hormone with neuroprotective effects, and its receptors can protect the brain against neurodegenerative diseases, emotional disorders, and cognitive decline [14, 15]. It has been demonstrated that estrogen deficiency in postmenopausal women may increase IL-6 production, and estrogen can inhibit the inflammatory response of astrocytes and microglia and downregulate inflammatory factors such as IL-6 and TNF-α [16]. Similarly, animal studiesfound that low serum levels of estradiol cause hippocampal inflammation, anxiety, and depression-like behaviors in mice, which were alleviated by estrogen supplementation [17]. Estrogen can also affect the IL-6/gp130 signaling pathway. Similarly, it has been observed that estradiol can inhibit IL-6 production and downregulate the expression of gp130 in secretory cells [18]. However, most of the previous studies only focused on the role of inflammation in MDD or emphasized the relationship between estrogen and MDD, with few studies exploring the mechanisms by which estrogen regulates inflammation and affects MDD. This study provides a new horizon in the field of MDD. Rebalancing estrogen levels may regulate IL-6 and sgp130 production as a new strategy for treating MDD. Considering the relationship between estrogen, inflammation, and depression, we hypothesized that estradiol can regulate the IL-6/sIL-6R/sgp130 signaling pathway and improve MDD. 

## Methods

### Participants

This study was a retrospective study with 131 inpatients who visited the Department of Psychiatry and Clinical Psychology of Renmin Hospital of Wuhan University from November 2021 to February 2023. Of 131 femal patients with MDD, 14 were excluded, 8 could not confirm whether they had a family history of mental illness, and 6 refused to participate. Finally, 117 female patients with MDD aged 42 (20–57) years were included in the analysis. At the same time, 122 healthy subjects aged 40 (30.75–56) years were recruited in this study. All patients with MDD underwent DSM-IV (SCID) structured clinical interviews before inclusion. In addition, the severity of depressive symptoms was assessed by a trained medical psychologist using the 24-item Hamilton Depression Scale (HAMD) [19]. Exclusion criteria were as follows: 1) taking antipsychotic drugs in the past three months; 2) suffering from other mental diseases, such as schizophrenia, obsessive–compulsive disorder and anxiety disorder; 3) suffering from diabetes, premenstrual Syndrome (PMS), malignant tumors, autoimmune diseases, serious heart or kidney diseases, etc. This study was approved by the Medical Ethics Review Committee of Renmin Hospital of Wuhan University (WDRY2021-K041). All subjects provided written informed consent.

### Collection and testing of blood samples

Blood samples were collected between the third and fifth days of the menstrual cycle [20]. All subjects were fasted at least for 8 h, the night before the examination and underwent venipathesis (about 3 mL) in the morning of the following day. The collected blood samples were centrifuged for 15 min at 3500 rpm/min at room temperature, and isolated serum samples were frozen at -80 ℃ until detection.

High-density lipoprotein cholesterol (HDL-C), uric acid (UA), creatinine (Cr), total cholesterol (TC), triglyceride (TG), urea, and low-density lipoprotein cholesterol (LDL-C) were detected by ADVIA 2400 automatic biochemical analyzer (Siemens, Germany). White blood cell counts and serum estradiol levels were measured using the Sysmex XN-20 system (Japan) and Siemens ADVIA Centaur CP (Germany), respectively. The serum levels of human serum IL-6, sgp130, and sIL-6Rα were detected by enzyme-linked immunosorbent assay (ELISA) kits (Quantikine DY206, DY227, DY008, and DY228; R&D Systems, USA). The serum levels of IL-6 were measured without diluting the sample, but the serum levels of sgp130 and serum sIL-6Rα were measured after diluting the samples 100 times.

### Statistical analysis

SPSS 22.0 and GraphPad Prism 7.0 were used for analyzing data and drawing graphs. LDL-C, WBC, and TC were analyzed using an independent sample t-test (all expressed as mean ± SD). Categorical variables (e.g., smoking, etc.) were compared using the Chi-square test. Age, Urea, Cr, TG, UA, HDL-C, E2, IL-6, sgp130, TC/HDL-C, and sIL-6Rα were analyzed using the Mann–Whitney U test (all expressed as median (P25, P75)). Spearman correlation and multiple linear regression were used to analyze the correlations between IL-6, sIL6Rα, sgp130, and HAMD. Receiver operating characteristic (ROC) curve was used to evaluate the diagnostic value of relevant indicators for MDD. In addition, the prediction probability and *P* value of multi-indicator ROC detection were determined by binary logistic regression analysis. In the binary logistic regression model, the grouping was used as the dependent variable, IL-6,sIL-6R,sgp130 and E2 as the covariables, and the prediction probability was further calculated using SPSS 22.0 software. Two-tailed *P* < 0.05 showed statistically significant differences.

## Results

### Study population

Table 1 presents basic clinical data for all participants. There was no difference in alcohol consumption, smoking, or age between the MDD group and the healthy control group (all *P* > 0.05). The serum levels of TC, E2, LDL-C, and sgp130 were significantly lower in the MDD group than in the healthy control group (all *P* < 0.05). The serum levels of WBC, IL-6, and sIL-6Rα in the MDD group were significantly higher than that of the control group (all *P* < 0.05).

**Table 1:** Comparison of baseline data between control group and MDD patients

Variable	Controls (*n* = 122)	MDD patients (*n* = 117)	Statistics	*P*
Age, year	40.00 (30.75–56.00)	42.00 (20.00–57.00)	*Z* = -1.026	0.305
Alcohol consumption (%)	5 (4.10)	6 (5.13)	*χ*^*2*^ = 0.144	0.704
Smoking (%)	6 (4.92)	8 (6.84)	*χ*^*2*^ = 0.399	0.528
Traumatic life events (%)	-	11 (9.40)	-	-
Family history of depression (%)	-	12 (10.26)	-	-
HAMD-24 score	-	22.00 (18.00–26.00)	-	-
Urea (mmol/L)	4.69 (3.96–5.72)	4.57 (3.77–5.27)	*Z* = -1.165	0.244
Cr (µmol/L)	53.00 (48.75–58.00)	51.00 (47.00–57.00)	*Z* = -0.778	0.437
UA (µmol/L)	286.00 (249.00–326.25)	277.00 (236.50–328.50)	*Z* = -0.698	0.485
WBC (10^9^/L)	5.49 ± 1.08	5.86 ± 1.42	*t* = -2.268	0.024
TC (mmol/L)	4.52 ± 0.81	4.23 ± 0.81	*t* = 2.751	0.006
TG (mmol/L)	0.99 (0.76–1.37)	1.03 (0.72–1.37)	*Z* = -0.102	0.919
HDL-C (mmol/L)	1.31 (1.12–1.54)	1.22 (1.03–1.47)	*Z* = -1.891	0.059
LDL-C (mmol/L)	2.54 ± 0.63	2.35 ± 0.69	*t* = 2.212	0.028
TC/HDL-C	3.31 (2.80–3.94)	3.26 (2.78–4.06)	*Z* = -0.025	0.980
IL-6 (pg/mL)	7.05 (4.69–12.03)	23.35 (17.92–44.97)	*Z* = -10.272	< 0.001
sIL-6Rα (ng/mL)	6.79 (2.30–11.54)	9.56 (5.62–13.21)	*Z* = -3.273	0.001
sgp130 (ng/mL)	80.93 (67.74–107.83)	52.05 (38.56–71.86)	*Z* = -7.546	< 0.001
E2 (pg/mL)	43.30 (19.84–78.75)	32.85 (18.09–57.87)	*Z* = -2.131	0.033

*MDD* Major depressive disorder, *Cr* creatinine, *UA* uric acid, *HAMD-24 score* 24-item Hamilton Depression Scale, *LDL-C* low-density lipoprotein cholesterol, *TG* triglyceride, *WBC* white blood cell, *TC* total cholesterol, *E2* estradiol, *sgp130* soluble glycoprotein 130, *IL-6* interleukin-6, *HDL-C* high-density lipoprotein cholesterol, *sIL-6Rα* soluble interleukin-6 receptor α

### Correlation between serum IL-6, sIL-6Rα, sgp130 and E2

**Fig. 1:**
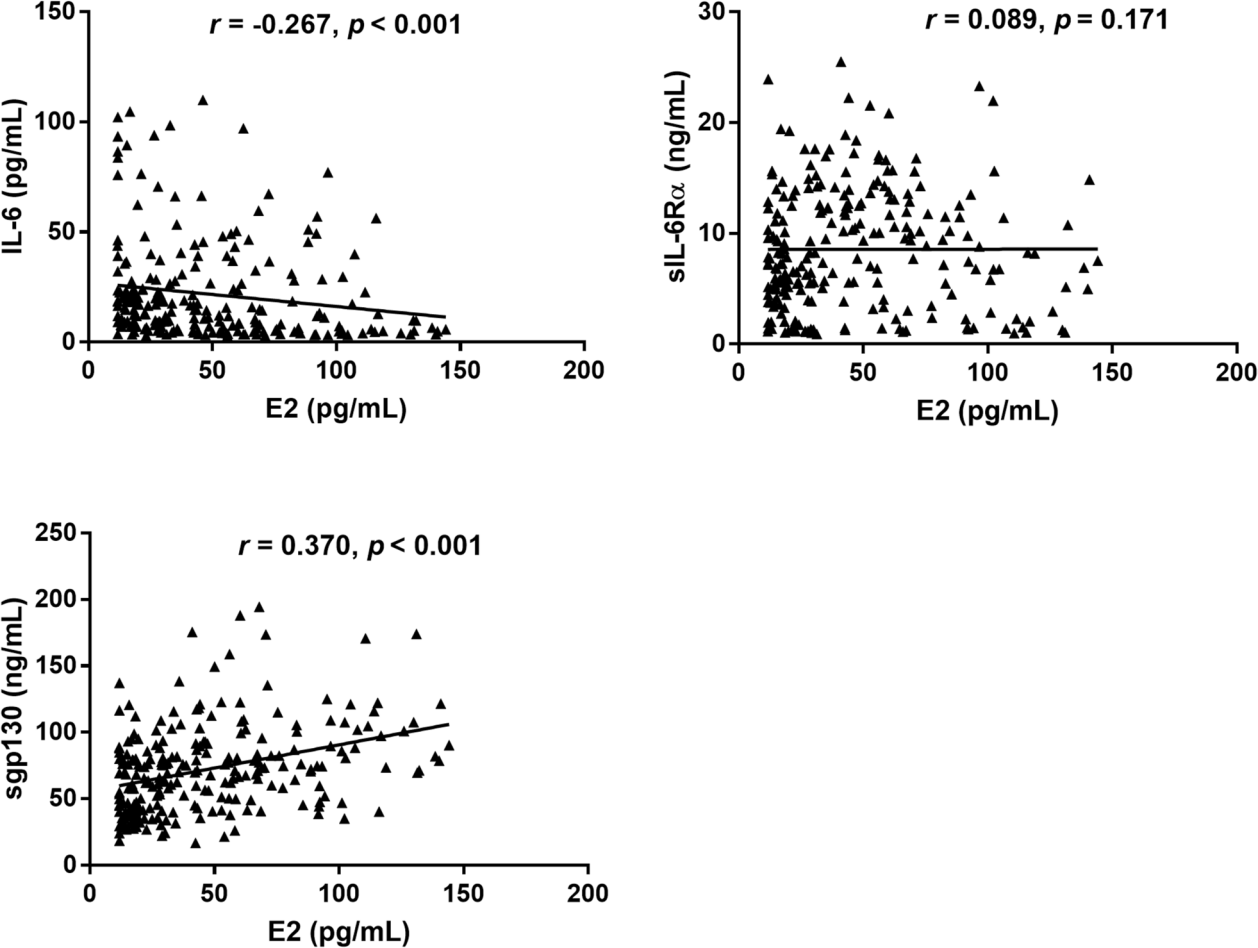
Spearman correlation analysis between IL-6, sIL-6Rα, sgp130 and serum E2

As shown in Table 2, after adjustment for smoking, TC, TG, alcohol consumption, TC/HDL-C, urea, Cr, UA, WBC, HDL-C, and LDL-C, IL-6 level was negatively correlated with the E2 level (*r* = -0.207, *P* = 0.001), but sgp130 level was positively correlated with the E2 level (*r* = 0.325, *P* < 0.001). There was no relationship between sIL-6Rα and E2 (*r* = -0.039, *P* = 0.540). Besides, spearman correlation analysis also showed that low serum levels of E2 were associated with high serum levels of IL-6 and low serum levels of sgp130 (*r* = -0.267, 0.370; *P* < 0.001) (Fig. 1).

**Table 2:** Multivariate linear regression to identify associations of IL-6, sIL-6Rα and sgp130 with E2

	(Constant)	IL-6	sIL-6Rα	sgp130
Standardized β-coefficien		-0.207	-0.039	0.325
*t*	4.678	-3.336	-0.614	5.613
*p*	< 0.001	0.001	0.540	< 0.001

After adjustment for smoking, TC, TG, alcohol consumption, urea, Cr, UA, TC/HDL-C, WBC, HDL-C and LDL-C

*Cr* creatinine, *UA* uric acid, *TC* total cholesterol, *HDL-C* high-density lipoprotein cholesterol, *WBC* white blood cell, *TG* triglyceride, *E2* estradiol, *IL-6* interleukin-6, *LDL-C* low-density lipoprotein cholesterol, *sgp130* soluble glycoprotein 130, *sIL-6Rα* soluble interleukin-6 receptor α

### Correlation between HAMD-24 score and serum levels of IL-6, sIL-6Rα, sgp130, and E2

**Fig. 2:**
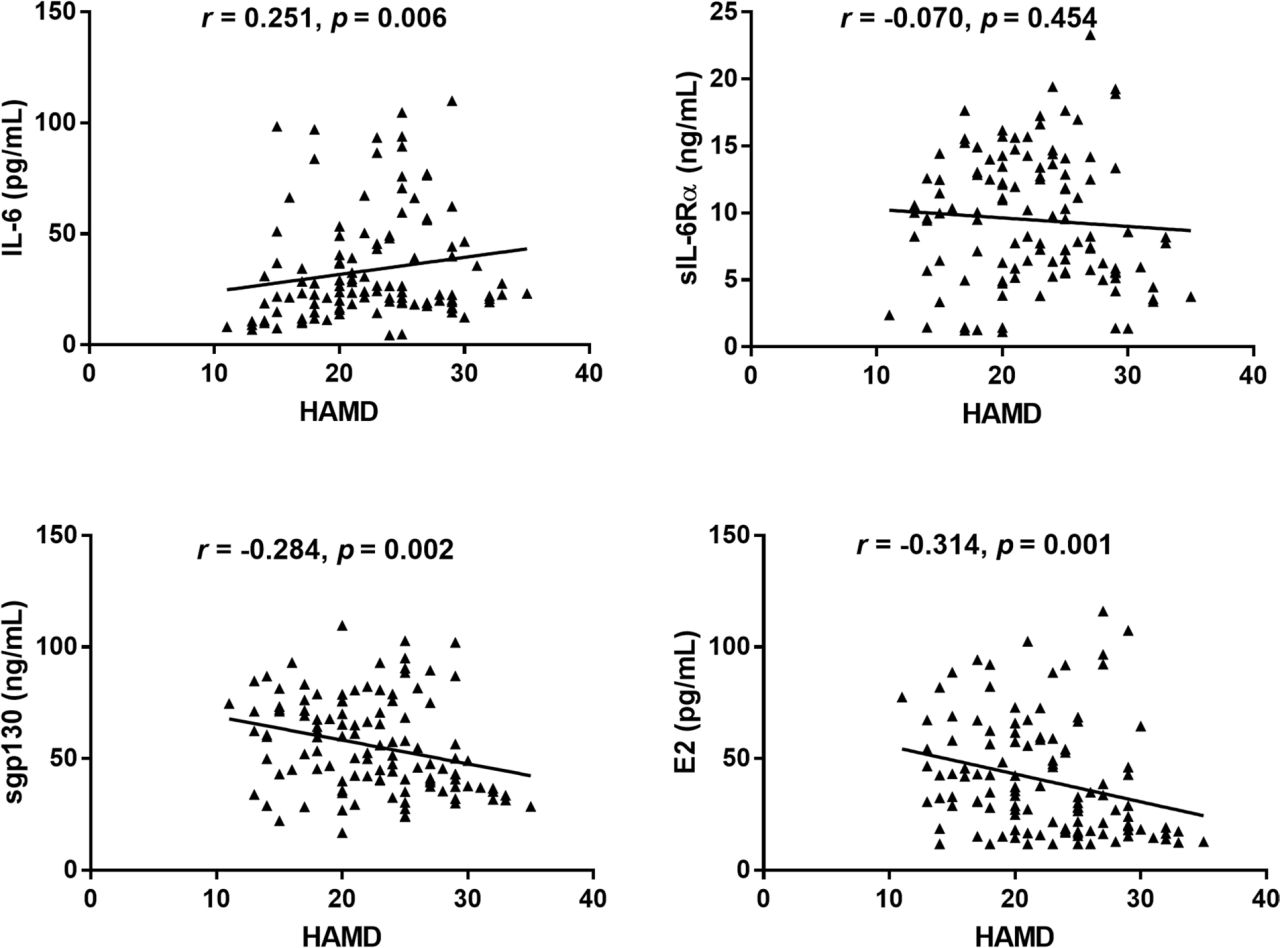
Correlations of IL-6, sIL-6Rα, sgp130, and E2 with HAMD-24 scores in MDD patients

We used multiple linear regression and spearman correlation to explore the relationship between the severity of MDD and serum levels of IL-6, sIL-6Rα, sgp130, and E2. In the case of multiple linear regression analysis without adjusting any variables, the score of HAMD-24 score was inversely associated with the serum level of sgp130 and E2 (*r* = -0.069 and -0.054, respectively, both *P* < 0.01). After adjustment for smoking, traumatic life events, alcohol consumption, and family history of depression in model 1, the HAMD-24 score was positively correlated with IL-6 level (*r* = 0.043, *P* < 0.05), and negatively correlated with sgp130 and E2 levels (*r* = -0.063 and -0.048, respectively, both *P* < 0.05). After additional adjustment for urea, Cr, UA, TC, TG, TC/HDL-C, WBC, HDL-C, and LDL-C in Model 2, the correlation between the above indicators remained significant (*r* = 0.047, -0.070, and -0.061, respectively; *P* < 0.05) (Table 3). In addition, the HAMD-24 score had a positive correlation with IL-6 level and a negative correlation with sgp130 and E2 levels (*r* = 0.251, -0.284, and -0.314, respectively; *P* < 0.01) (Fig. 2).

**Table 3:** Multiple linear regression analysis of the correlation between the levels of IL-6, sIL-6Rα, sgp130, E2 and the scores of HAMD-24 in MDD patients

Parameter	Unadjusted,β, *P* value (95% CI)	Adjusted,β, *P* value (95% CI)	
		Model 1	Model 2
IL-6	0.037, 0.070 (-0.003 to 0.076)	0.043, 0.035 (0.003 to 0.082)	0.047, 0.036 (0.003 to 0.092)
sIL-6Rα	-0.077, 0.454 (-0.280 to 0.126)	-0.070, 0.497 (-0.273 to 0.133)	-0.050, 0.650 (-0.270 to 0.169)
sgp130	-0.069, 0.003 (-0.115 to -0.024)	-0.063, 0.009 (-0.109 to -0.016)	-0.070, 0.012 (-0.125 to -0.015)
E2	-0.054, 0.005 (-0.091 to -0.017)	-0.048, 0.013 (-0.086 to -0.010)	-0.061, 0.009 (-0.107 to -0.015)

Model 1 adjusted for smoking, traumatic life events, alcohol consumption and family history of depression. Model 2 for the same variables as Model 1 as well as urea, Cr, UA, TC, TG, TC/HDL-C, WBC, HDL-C and LDL-C

*95% CI* 95% confidence interval, *Cr* creatinine, *UA* uric acid, *TC* total cholesterol, *HDL-C* high-density lipoprotein cholesterol, *WBC* white blood cell, *TG* triglyceride, *E2* estradiol, *IL-6* interleukin-6, *LDL-C* low-density lipoprotein cholesterol, *sgp130* soluble glycoprotein 130, *sIL-6Rα* soluble interleukin-6 receptor α

### Association of IL-6, sIL-6Rα, sgp130, and E2 levels with the risk factors of MDD

Spearman correlation analysis was used to investigate the relationship between other risk factors of MDD and IL-6, sIL-6Rα, sgp130, and E2 levels. IL-6 level was positively correlated with WBC (*r* = 0.202, *P* = 0.029), and negatively correlated with TC and LDL-C levels (*r* = -0.239 and -0.187, respectively; both *P* < 0.05) (Table 4). sgp130 was negatively associated with the serum levels of urea, TC, TG, LDL-C, and TC/HDL-C (*r* = -0.250, -0.372, -0.392, -0.287, and -0.283, respectively; all *P* < 0.01). The serum level of E2 was negatively associated with the serum levels of urea, TC, TG, LDL-C and TC/HDL-C (*r* = -0.187, -0.458, -0.389, -0.365, and -0.326, respectively; all *P* < 0.05).

**Table 4:** Correlation analysis of IL-6, sIL-6Rα, sgp130 and E2 with risk factors of depression in MDD patients

Risk factor	IL-6		sIL-6Rα		sgp130		E2	
	*r*	*p*	*r*	*p*	*r*	*p*	*r*	*p*
Urea	-0.026	0.785	-0.133	0.154	-0.250	0.006	-0.187	0.044
Cr	-0.180	0.052	-0.163	0.079	-0.099	0.289	-0.105	0.261
UA	0.090	0.336	0.109	0.241	0.161	0.084	-0.001	0.990
WBC	0.202	0.029	0.112	0.228	0.069	0.459	0.057	0.543
TC	-0.239	0.009	-0.111	0.233	-0.372	< 0.001	-0.458	< 0.001
TG	-0.170	0.067	-0.131	0.159	-0.392	< 0.001	-0.389	< 0.001
HDL-C	-0.141	0.129	-0.007	0.937	0.015	0.875	-0.012	0.896
LDL-C	-0.187	0.043	-0.065	0.488	-0.287	0.002	-0.365	< 0.001
TC/HDL-C	-0.055	0.555	-0.081	0.385	-0.283	0.002	-0.326	< 0.001

*MDD* Major depressive disorder, *Cr* creatinine, *UA* uric acid, *TC* total cholesterol, *HDL-C* high-density lipoprotein cholesterol, *WBC* white blood cell, *TG* triglyceride, *E2* estradiol, *IL-6* interleukin-6, *LDL-C* low-density lipoprotein cholesterol, *sgp130* soluble glycoprotein 130, *sIL-6Rα* soluble interleukin-6 receptor α

### Prognostic values of IL-6, sIL-6Rα, sgp130 and E2 in patients with MDD

**Fig. 3:**
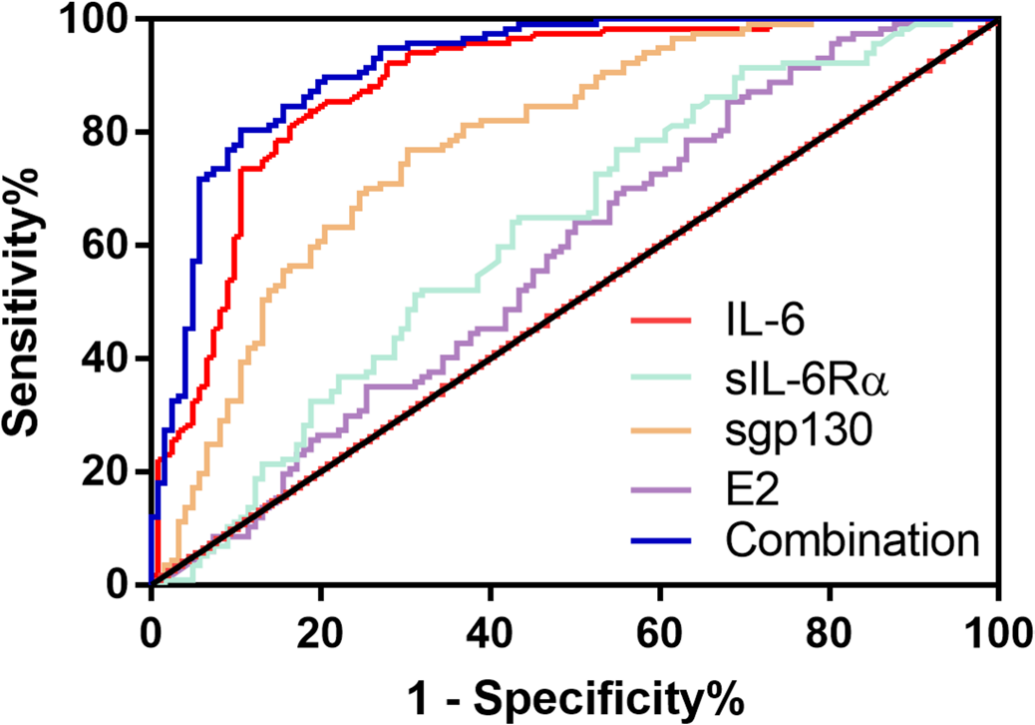
ROC curve of combined diagnosis of MDD with IL-6, sIL-6Rα, sgp130 and E2. 95% CI: 95% confidence interva; AUC: area under the receiver operating characteristic curve; E2: estradiol; IL-6: interleukin-6; sgp130: soluble glycoprotein 130; sIL-6Rα: soluble interleukin-6 receptor α

The ROC curve of the prognostic value of IL-6, sIL6Rα, sgp130, and E2 for MDD is shown in Fig. 3. AUC comparison: Combination (0.919) > IL-6 (0.885) > sgp130 (0.783) > sIL-6Rα (0.622) > E2 (0.580). Sensitivity comparison: IL-6 (85.47%) > Combination (80.34%) > sIL-6Rα (76.92%) = sgp130 (76.92%) > E2 (62.39%). Specificity comparison: Combination (89.34%) > IL-6 (79.51%) > sgp130 (69.67%) > E2 (50.82%) > sIL-6Rα (45.08%). Youden index comparison: Combination (0.6968) > IL-6 (0.6498) > sgp130 (0.4659) > sIL-6Rα (0.2200) > E2 ( 0.1312). The cut-off values of IL-6, sIL-6Rα, sgp130, and E2 were 13.57 pg/L, 5.60 ng/mL, 73.33 ng/mL, and 42.85 pg/mL, respectively (Table 5).

**Table 5:** ROC curve analysis of MDD diagnosis by IL-6, sIL-6Rα, sgp130 and E2

	Sensitivity (%)	Specificity (%)	AUC	95%CI	Youden index	Cut-off
IL-6	85.47	79.51	0.885	0.841 to 0.928	0.6498	13.57 pg/mL
sIL-6Rα	76.92	45.08	0.622	0.551 to 0.693	0.2200	5.60 ng/mL
sgp130	76.92	69.67	0.783	0.724 to 0.841	0.4659	73.33 ng/mL
E2	62.39	50.82	0.580	0.507 to 0.652	0.1312	42.85 pg/mL
Combination	80.34	89.34	0.919	0.884 to 0.954	0.6968	-

*IL-6* interleukin-6, *E2* estradiol, *sgp130* soluble glycoprotein 130, *sIL-6Rα* soluble interleukin-6 receptor α

## Discussion

This case–control study reported the evidence of associations between the IL-6/sIL-6R/sgp130 complex and E2 in patients with MDD. Firstly, we found that female patients with MDD have higher serum levels of IL-6 and sIL-6Rα, and lower serum levels of sgp130 and E2. Secondly, E2 levels were negatively correlated with IL-6 levels and positively correlated with sgp130 levels. In addition, high levels of IL-6 and low levels of sgp130 and E2 were associated with higher 24-item Hamilton scores. Thirdly, we demonstrated that the IL-6/sIL-6R/sgp130 complex has a certain diagnostic prognostic value in MDD, and the combination of several indicators can significantly improve the prognostic value.

MDD is considered, in some sense, to be a chronic inflammatory disease with altered serum levels of cytokines [21, 22]. IL-6, a common inflammatory factor, is involved in the pathogenesis of MDD through various mechanisms. IL-6 can cross the blood–brain barrier (BBB)and increase synaptic contractility by directly affecting neurons or regulating microglia and other immune cells, thereby aggravating depression [23, 24]. Secondly, IL-6 can directly affect brain function and neurotransmitter production, leading to the progression and poor prognosis of MDD [25]. In addition, IL-6 can inhibit hippocampal neurogenesis in depression by acting on IL-6 receptors or a transmembrane protein, gp130, in the dentate gyrus [26, 27] or by stimulating the hypothalamic–pituitary–adrenal (HPA) axis [27, 28]. Impaired neurogenesis is an important mechanism in MDD [29]. Consistent with our results, the meta-analysis by Dahl et al. confirmed that IL-6 levels are significantly higher in patients with MDD than in the healthy control group [30]. IL-6 can induce microglial cell activation [31], oxidative stress, and neuronal apoptosis [32], and HPA axis dysfunction [33], and reduce the secretion of norepinephrine and 5-hydroxytryptamine [34], ultimately leading to MDD. In addition, Mao [35] and Roohi [36] et al. demonstrated that IL-6 is an important inflammatory factor that exacerbates MDD and is positively correlated with the severity and prognosis of MDD. Animal studies have also shown that elevated levels of central or peripheral IL-6 may be associated with depressive symptoms [24].

Spg130, a natural inhibitor of the IL-6 trans-signaling pathway, has been shown to be closely related to MDD as a potential therapeutic target [37]. Previous findings concerning sgp130 and its relationship with MDD are controversial. Sukoff et al. demonstrated that continuously increased levels of IL-6 in the central nervous system (CNS) can lead to depression-like phenotype in rodents, while sgp130 FC can significantly reduce IL-6 levels and improve depression-like behavior, suggesting the anti-inflammatory and antidepressant effects of sgp130 [38]. Besides, it has been shown that there is no significant difference in sgp130 levels in the cerebrospinal fluid between depressed and control groups [39]. The possible reason is that the sgp130 level in cerebrospinal fluid is significantly lower than that in serum, but the exact cause is unclear and more studies are needed.

In addition, numerous studies have illustrated a robust association between IL-6/sIL-6R/sgp130 complex and E2. As a steroid hormone with anti-inflammatory effects, estrogen downregulates the levels of IL-6 and gp130 levels in osteoblasts and breast cancer cells, thereby affecting the downstream IL-6/gp130 signaling pathway [39, 40]. Estradiol can also downregulate gp130 and IL-6 levels in mouse bone marrow, protecting against osteoporosis [40, 41]. However, the relationship between estrogen and the IL-6/sIL-6R/sgp130 complex has been less studied in patients with MDD. Notably, our results demonstrated that patients with MDD have low levels of E2. In particular, E2 levels were negatively correlated with IL-6 levels and positively correlated with sgp130 levels, indicating that estrogen exerts antidepressant effects by suppressing inflammation.. Therefore, balancing IL-6and sgp130 levels with estrogen may be crucial for improving depressive symptoms.

However, our study has certain limitations. Firstly, our study was a case–control study and could not determine causal relationships. Secondly, although this study adjusted the effects of many variables, other confounding factors were not excluded. Thirdly, the sample size of this study was small. Future studies with large sample sizes are needed to further explore the relationship between estrogen and the IL-6/sIL-6R/sgp130 complex in patients with MDD. Finally, in terms of the molecular mechanism, sevel studies have shown that the protein expression [38] and mRNA level of IL-6 are significantly increased in the peripheral blood of patients with MDD [42]. In addition, changes in IL6 DNA methylation may also be closely related to the development of MDD [43]. However, it is still unclear whether altered IL-6 protein and gene expression are related to E2, and the effect of sgp130 on MDD is currently limited to the serological studies. Therefore, more in-depth studies on the altered protein and gene expression of IL-6 and sgp130 are needed in patients with MDD to unravel the relationship between E2 and the above mechanisms.

In conclusion, our findings demonstrated that the serum levels of E2 in patients with MDD are closely related to the IL-6/sIL6R/sgp130 complex. We found that IL-6 and sgp130 have potential diagnostic value for MDD, providing a deeper insight into the pathogenesis of MDD.

## Data Availability

The datasets used during the current study are available from the corresponding author according to reasonable requirements.

## Abbreviations

MDD: Major depressive disorder
HAMD-24: 24-Item Hamilton Depression Scale
UA: Uric acid
Cr: Creatinine
HDL-C: High-density lipoprotein cholesterol
sgp130: Soluble glycoprotein 130
sIL-6Rα: Soluble interleukin-6 receptor α
IL-6: Interleukin-6
LDL-C: Low-density lipoprotein cholesterol
TG: Triglyceride
WBC: White blood cell
TC: Total cholesterol
E2: Estradiol
